# Unplanned pregnancy and perinatal depression: secondary exploratory analyses from a racially and ethnically diverse, low-income sample of birthing people in the United States

**DOI:** 10.1186/s12884-025-08009-w

**Published:** 2025-08-21

**Authors:** Katherine A. Czech, Jaime Hamil, Bayley J. Taple, Jody D. Ciolino, Ann Kan, Allison M. Letkiewicz, Alicia Diebold, S. Darius Tandon

**Affiliations:** 1 Department of Psychology, University of Denver, 2155 S Race St, Denver, CO 80210 USA; 2 Institute for Public Health and Medicine, Center for Community Health, Feinberg School of Medicine, Northwestern University, 750 N. Lake Shore Dr, Chicago, IL 60611 USA; 3 Center for Behavioral Intervention Technologies, Feinberg School of Medicine, Northwestern University, 750 N. Lake Shore Dr, Chicago, IL 60611 USA; 4 Division of Biostatistics and Informatics, Department of Preventive Medicine, Feinberg School of Medicine, Northwestern University, 680 N Lake Shore Drive, Suite 1400, Chicago, IL 60611 USA; 5 Department of Psychiatry and Behavioral Sciences, Feinberg School of Medicine, Northwestern University, 680 N. Lakeshore Drive, Chicago, IL 60611 USA

**Keywords:** Unplanned pregnancy, Perinatal depression, Maternal mental health, Perinatal mental health

## Abstract

**Background:**

The association between unplanned pregnancy and perinatal depression is understudied in racially and ethnically diverse and low-income populations. The present study was compromised of a secondary analysis of data from a low-income and 70% racially and ethnically minoritized sample to examine associations between unplanned pregnancy and perinatal depressive symptoms and to explore potential moderation by social factors.

**Methods:**

Pregnant individuals (*n* = 808) were enrolled in a larger study evaluating the effectiveness of a preventative intervention for postpartum depression, and self-reported depressive symptoms were collected prenatally and at 12 weeks postpartum using the 16-item Quick Inventory of Depressive Symptomatology. Multiple linear regression examined the relationship between unplanned pregnancy and maternal depressive symptoms, and the potential interactions between unplanned pregnancy and (1) race/ethnicity (2), education level (3), first-time parent status, and (4) a prenatal mental healthcare utilization. Analyses were conducted both within the sample as a whole and within racial/ethnic subgroups.

**Results:**

While bivariate regression revealed a significant association between unplanned pregnancy and prenatal depressive symptoms (β = 0.88, 95% CI [0.27, 1.49], *p* = 0.005), unplanned pregnancy was not significantly associated with prenatal or postpartum depression in adjusted models in the full sample. Analyses suggested different trends in racial/ethnic subgroups. Specifically, endorsing prenatal mental healthcare utilization and unplanned pregnancy was associated with higher prenatal depressive symptoms in the Latine subgroup compared to those whose pregnancies were planned (β = 4.59, 95% CI [0.60, 8.59], *p* = 0.025). Additionally, unplanned pregnancy was associated with higher depressive symptoms at 12 weeks postpartum compared to those with planned pregnancy also in the Latine sample (β = 1.06; 95% CI [0.10, 2.03], *p* = 0.031). Unplanned pregnancy was not found to be associated with prenatal depressive symptoms in the adjusted models of any other racial/ethnic subgroups.

**Conclusions:**

These secondary analyses from a larger study suggest potentially important differences in the association between unplanned pregnancy and perinatal depressive symptoms by racial/ethnic subgroups. Future research should acknowledge the myriad stressors and protective factors experienced by low-income and racially and ethnically diverse perinatal populations when evaluating differences in outcomes among racial/ethnic subgroups.

**Supplementary Information:**

The online version contains supplementary material available at 10.1186/s12884-025-08009-w.

## Introduction

Perinatal depression, defined as depression occurring during the prenatal and/or postpartum periods, is estimated to have a prevalence of 11.9% globally [1], with evidence to suggest that the prevalence of perinatal depression and anxiety disorders is on the rise in the United States [2]. Previous studies suggest perinatal depression has profound implications on the well-being of pregnant individuals and their children. For example, one study suggests women with perinatal mood and anxiety disorders (PMAD) have a higher incidence of preterm delivery compared to those without PMAD or serious mental illness [9.7 (95% CI 9.4–10.0) per 100 deliveries compared to 6.7 (95% CI 6.7–7.0) per 100 deliveries] [2]. Additionally, those with PMAD have a higher incidence of severe maternal morbidity and mortality compared to those without PMAD or serious mental illness [2.3 (95% CI 2.2–2.4) per 100 deliveries compared to 1.5 (95% CI 1.5–1.5) per 100 deliveries] [2]. Further, findings suggest that the offspring of individuals who experience perinatal depression are at increased risk for mental health problems [3], with one meta-analysis finding a 70% increase in the odds of developing depression among adolescent and adult offspring of mothers who had perinatal depression [1.70 (95% CI, 1.60–2.65)] [4]. Therefore, understanding the risk factors of perinatal depression and their consequences is paramount.

Unplanned pregnancy is one such risk factor for perinatal depression that has not been sufficiently explored [5]. According to the United States Centers for Disease Control and Prevention, it is estimated that 41.6% of pregnancies in the United States in 2019 were unintended [6], suggesting it is a prevalent phenomenon worthy of inquiry. To note, the terms ‘unplanned’, ‘unintended’, and ‘unwanted’ pregnancy are often used interchangeably [5]. A sensitivity analysis in a recent meta-analysis indicated that pregnancy planning had a similar effect on perinatal depressive symptoms regardless of it being characterized as unwanted versus unintended or unplanned [7]. In this paper, we will use the term “unplanned pregnancy.”

The context surrounding unplanned pregnancy may place individuals at increased risk for experiencing perinatal depression. For example, the disruption to career, educational, and other life pursuits, as well as increased financial strain, often associated with an unplanned pregnancy may evoke depressive symptoms [8]. Moreover, receiving low social support, which is a well-documented risk factor for perinatal depression [9, 10], has been found to be more strongly associated with unplanned pregnancy compared to planned pregnancy [11]. Indeed, one study of Iranian birthing people found that the emotional reactions of romantic partners to an unplanned pregnancy mediated the relationship between unplanned pregnancy and maternal prenatal mental health. Another study found that those with lower relationship quality were more likely to experience psychological distress in the context of unplanned pregnancy [12]. Further, pre-pregnancy stress and depressive symptoms, which are also risk factors for perinatal depression, have been found to be associated with unplanned pregnancy [13], likely mediated by lower adherence to effective contraceptive methods [14].

Pooled evidence suggests that unplanned pregnancy is generally associated with maternal depression [5, 7]. However, these estimates include studies with samples from mostly White and highly educated populations, as well as from studies conducted in countries outside the United States. Further, the authors of a recent meta-analysis noted that they were not able to obtain pooled effects of pregnancy intention on perinatal depression by demographic subgroups due to most studies only reporting results based on the full sample [7]. While these pooled data are valuable, there is a dearth of research that has studied associations between unplanned pregnancy and maternal depression in racially and ethnically diverse and low-income samples in the United States.

This represents an important area of study given that significant disparities in rates of perinatal depression and unplanned pregnancy exist along ethnic and racial lines. A 2019 review from the Centers for Disease Control and Prevention reported that Hispanic birthing persons experience rates of unintended pregnancy of 38.8 per 1,000 women, non-Hispanic Black birthing persons experience rates of unintended pregnancy of 63.2 per 1,000 women, and non-Hispanic White birthing persons experience rates of unintended pregnancy of 28.2 per 1,000 women [6]. It’s critical to note that these disparities are not inherent to individual identities, and that they are better explained by systemic factors, such as socioeconomic status, education attainment, and access to health insurance, as well as other social ecological factors, such as age and relationship status [15]. Regarding perinatal depression, non-Hispanic Black and Latine samples have been shown to experience higher rates of prenatal [16] and postpartum [17] depression. Systemic factors also contribute to these disparities, as these communities disproportionately experience institutionalized stressors, such as medical racism, racist policing practices, economic inequality, and financial insecurity [18–20], which are important to consider in the context of perinatal mental health. Additionally, there is consistent evidence that individuals minoritized by race and ethnicity receive substantially lower quality postpartum mental healthcare compared to their White counterparts [21, 22]. Consequently, research is needed to understand how unplanned pregnancy may be associated with perinatal depression in the unique and understudied contexts of racially and ethnically diverse individuals.

Further, other contextual factors may be salient in better understanding the association between pregnancy planning and perinatal depression in minoritized racial and ethnic populations [7]. Education [23], first-time parent status [24], and factors associated with pre-pregnancy mental health [25, 26], have been associated with perinatal depression and therefore, may moderate the relationship between pregnancy planning and maternal depression in these populations.

To address these gaps in the literature, the present study sought to garner a more robust understanding of the association between pregnancy planning and perinatal depression in a low-income, racially and ethnically diverse sample of birthing people. Specifically, the aims of this secondary analysis were to (1) examine the association between pregnancy planning and depression during the prenatal period, (2) secondarily examine this relationship in the presence of potential confounders (e.g., race/ethnicity, education, first-time parent status, language, prenatal mental healthcare utilization ), and (3) evaluate the interaction effects of pregnancy planning and each of the following demographic characteristics on maternal depression: race/ethnicity, education level, first-time parent status, and prenatal mental healthcare utilization. These analyses were repeated using maternal depression scores at 12 weeks postpartum. Additionally, subgroup analyses were conducted within individual racial/ethnic subgroups to better understand the unique experiences of individuals within each group.

## Methods

### Procedure and participants

The present study was a secondary analysis of archival data from a cluster-randomized trial evaluating the effectiveness of Mothers and Babies (MB), a group-based intervention for the prevention of postpartum depression. The parent study evaluated differences in mental health outcomes for parents participating in home visiting services between groups from the three following study conditions: MB delivered by mental health professionals, MB delivered by paraprofessionals, and usual home visiting services. Home visiting services are programs in which families, who often reside in communities with low income, receive assistance during regularly scheduled visits from trained home visiting paraprofessionals who provide resources and help improve the family’s overall health and well-being. The primary study design and overall findings have been published elsewhere [27, 28].

Briefly, data collection occurred from January 2017 through July 2019. Participants were recruited from home visiting programs in Illinois, Michigan, Ohio, Minnesota, Missouri, Iowa, and West Virginia, and were considered eligible if they were 16 years of age or older, pregnant at a gestational age no greater than 33 weeks, and spoke either English or Spanish as their primary language. Participants provided written or oral consent to participate and completed assessments at baseline, post-intervention, 12 weeks postpartum, and 24 weeks postpartum. Study data were collected and managed using REDCap (Research Electronic Data Capture) tools [29, 30]. The study received Institutional Review Board approval.

Baseline data collection occurred prior to intervention delivery and during the prenatal period (median weeks’ gestation was 23 weeks). For the 12 weeks postpartum analyses, participants were sent questionnaires to complete the assessment at 12 weeks postpartum and had approximately two months to complete this assessment. Participants who had missing data for one or more of the variables explored at baseline (*n* = 16) were excluded, leaving a prenatal analytic sample of *n* = 808. Participants who did not have data for one or more of the variables explored at baseline and 12-weeks postpartum (*n* = 128) were excluded, leaving a 12-weeks postpartum analytic sample of *n* = 696. Given that results from the cluster-randomized trial did not find significant differences between the study arms [28], we did not consider the study arm variable in these analyses, which participants received after baseline. Therefore, we grouped all participants together, regardless of intervention received for both prenatal and postpartum analyses for consistency.

### Measures

#### Outcome

Depression was assessed using the Quick Inventory of Depressive Symptomology (QIDS-SR16) [31], a continuous measure ranging from 0 to 27 points, where higher scores indicate a higher level of depressive symptoms. Depression was assessed during the prenatal period prior to intervention delivery and at 12 weeks postpartum. The QIDS-SR16 has previously demonstrated strong psychometric properties, particularly in samples from the United States [32], and has been used previously in a postpartum sample [33]. Reliability in the present sample was acceptable (Cronbach’s alpha = 0.74).

#### Unplanned pregnancy

The survey item assessing unplanned pregnancy: “Was your current pregnancy planned?” was asked at baseline during the prenatal period, prior to intervention delivery. For these analyses, unplanned was coded as “1,” and planned was coded as “0”.

#### Interaction terms and covariates

The additional variables of race/ethnicity, education level, first-time parent status, prenatal mental healthcare utilization, and primary language were all collected at baseline. To note, participants were only permitted to select one racial/ethnic identity when completing the demographics survey.

When conducting analyses on the full sample, we categorized minoritized race/ethnicity as a dichotomous variable, in which “Black/African American”, “Hispanic/Latina” (hereafter referred to as “Latine”), “Asian American”, “Native American”, and “Bi-racial” were considered “minoritized”, and “White/Caucasian” (hereafter referred to as “White”) comprised the “not minoritized” category. Given that collapsing demographic groups into one group (i.e. “minoritized race/ethnicity”) does not acknowledge the complexity and nuance afforded by the individuals in each group and may lead to overlooking important clinical differences between groups [34], we also conducted subgroup analyses by racial/ethnic group for those belonging to the Black/African American, Latine, and White subgroups. Subgroup size constraints precluded us from conducting analyses with the sample’s remaining racial and ethnic groups.

Level of educational attainment was converted into a dichotomous factor-level variable with levels of “no college” and “at least some college.” Participants who responded with a number larger than zero to the question, “How many other children have you given birth to?” were categorized as not first-time parents, and all who responded to this question with “zero” were categorized as first-time parents. Additionally, participants were categorized as either primary English or Spanish speakers.

The present study did not have pre-pregnancy depression variables; therefore, we used variables that might have been associated, such as endorsement of current utilization of therapy for depression and/or medication for depression during the prenatal period. This “prenatal mental healthcare utilization” variable was treated as dichotomous in analyses. While prenatal mental healthcare utilization is indeed not a measure of pre-pregnancy depression, we believe this measure captures valuable behaviors related to pre-pregnancy depression. There is evidence in the literature of pre-pregnancy mental healthcare utilization continuing into the prenatal period, with one study finding 62.6% of participants in a low-income sample who had been on combined psychotherapy and medication treatment pre-pregnancy continuing psychotherapy into the prenatal period [35]. Additionally, another study found that in a sample of pregnant people with a history of major depression, those who were in psychotherapy during the prenatal period were more likely to have had a psychotherapy or psychiatry session in the previous two years [36].

### Participant characteristics

Table 1 presents participant characteristics. Briefly, participants were enrolled during the prenatal period (median weeks’ gestation of 23 weeks). Approximately 70% of the present sample held identities that are minoritized by race/ethnicity, with 44.4% of the sample identifying as African American/Black, 30.3% identifying as White, 20% identifying as Latine, and 3.6% identifying as Bi-racial. Cell counts for participants identifying as Asian and Native American were too small to report. Additionally, 72% of the sample reported an annual income below $25,000. Just over 40% of the sample identified as having at least some college education, 36.9% identified as first-time parents, and 17.3% endorsed prenatal mental healthcare utilization for depression. Nearly 65% of the sample endorsed their pregnancies as being “unplanned,” and the prenatal QIDS-SR16 mean score was 8.01 (SD = 4.25).

**Table 1:** Participant characteristics for baseline (Prenatal) analysis

Overall *N*	808
Age: *Mean (SD)*	26.28 (5.84)
Minoritized race/ethnicity: N (%)	563 (69.7%)
African American/Black	359 (44.4%)
Asian	**
Bi-racial	29 (3.6%)
Latine	161 (20.0%)
Native American	**
White (not-minoritized)	245 (30.3%)
Unplanned pregnancy: N (%)	522 (64.6%)
Employed: N (%)	286 (36.2%)
Unknown	1 (0.001%)
No	517 (64.0%)
Part-time	167 (20.7%)
Full-time	123 (15.2%)
At least some college education: N (%)	329 (40.7%)
Income: N (%)	
Unknown	19 (2.4%)
<$25,000	582 (72.0%)
$25,000–$49,999	150 (18.6%)
$50,000–$74,999	32 (4.0%)
$75,000–$99,999	13 (1.6%)
$100,000 +	12 (1.5%)
Spanish language	110 (13.6%)
Weeks’ gestation: *Median (Range)*	23 (4–39)
Number of current children given birth to: *Median (Range)*	1 (0–9)
First-time parent: N (%)	298 (36.9%)
MDE*: N (%)	29 (3.6%)
Prenatal mental healthcare utilization (counseling and/or prescribed medication): N (%)	140 (17.3%)
Quick Inventory of Depressive Symptomology (QIDS-SR16): *Mean (SD)*	8.01 (4.25)

*MDE Major Depressive Episode

**To preserve anonymity, we do not report cell counts < 10

### Data analysis plan

The primary objective of this secondary analysis was to understand the relationship between unplanned pregnancy and depressive symptoms during the prenatal period for parents of singleton births. The cross-sectional analysis used a linear model for the continuous QIDS-SR16 outcome with intention of pregnancy as the predictor of interest. Adjusted models included the above pre-specified baseline covariates of minoritized race/ethnicity, first-time parent status, education level, prenatal mental healthcare utilization, and language. This analysis strategy closely mirrors that of the primary analyses in the parent study [28]. Four additional models were tested with each interaction term of minoritized race/ethnicity, first-time parent status, education level, and prenatal mental healthcare utilization, all of which included the covariates from the multiple regression model.

As a secondary objective, we sought to explore potential effects of pregnancy planning on postpartum depressive symptoms at 12 weeks postpartum. This analysis involved a linear model for continuous outcome (QIDS-SR16 at 12 weeks) with independent variables including: pregnancy planning, baseline (prenatal) QIDS-SR16 score, and the aforementioned baseline covariates. We further explored the same interactions as indicated above.

The primary and secondary objectives of these analyses were repeated in subgroup analyses separated by racial/ethnic group to determine if there were unique outcomes based on whether participants identified as Black/African American, Latine, or White.

Analyses were completed using R version 2024.9.1.394 [37], and all statistical tests assumed a two-sided 5% level of significance. There were no adjustments made for multiple hypothesis tests as these analyses were strictly exploratory in nature.

## Results

We first present the unadjusted association between unplanned pregnancy and prenatal depressive symptoms followed by the adjusted association in the full sample. Next, we present the adjusted association between unplanned pregnancy and depressive symptoms at 12 weeks postpartum also in the full sample. See “Additional File 1” for post hoc analyses related to the relationships among main study variables. Lastly, we present on adjusted associations between unplanned pregnancy and prenatal and postpartum depressive symptoms in the Latine, Black/African American, and White racial/ethnic subgroups. See “Additional File 2” for full results of the multiple linear regression models, including covariates, in the full sample and racial/ethnic subgroups. For interpretation of study findings, specific validated screening scores for the QIDS are as follows: 0–5 = no depression, 6–10 indicates mild depression, 11–15 indicates moderate depression, 16–20 indicates severe depression, ≥ 21 indicates very severe depression.

### Relationships among main study variables and prenatal depression in full sample

**Fig. 1:**
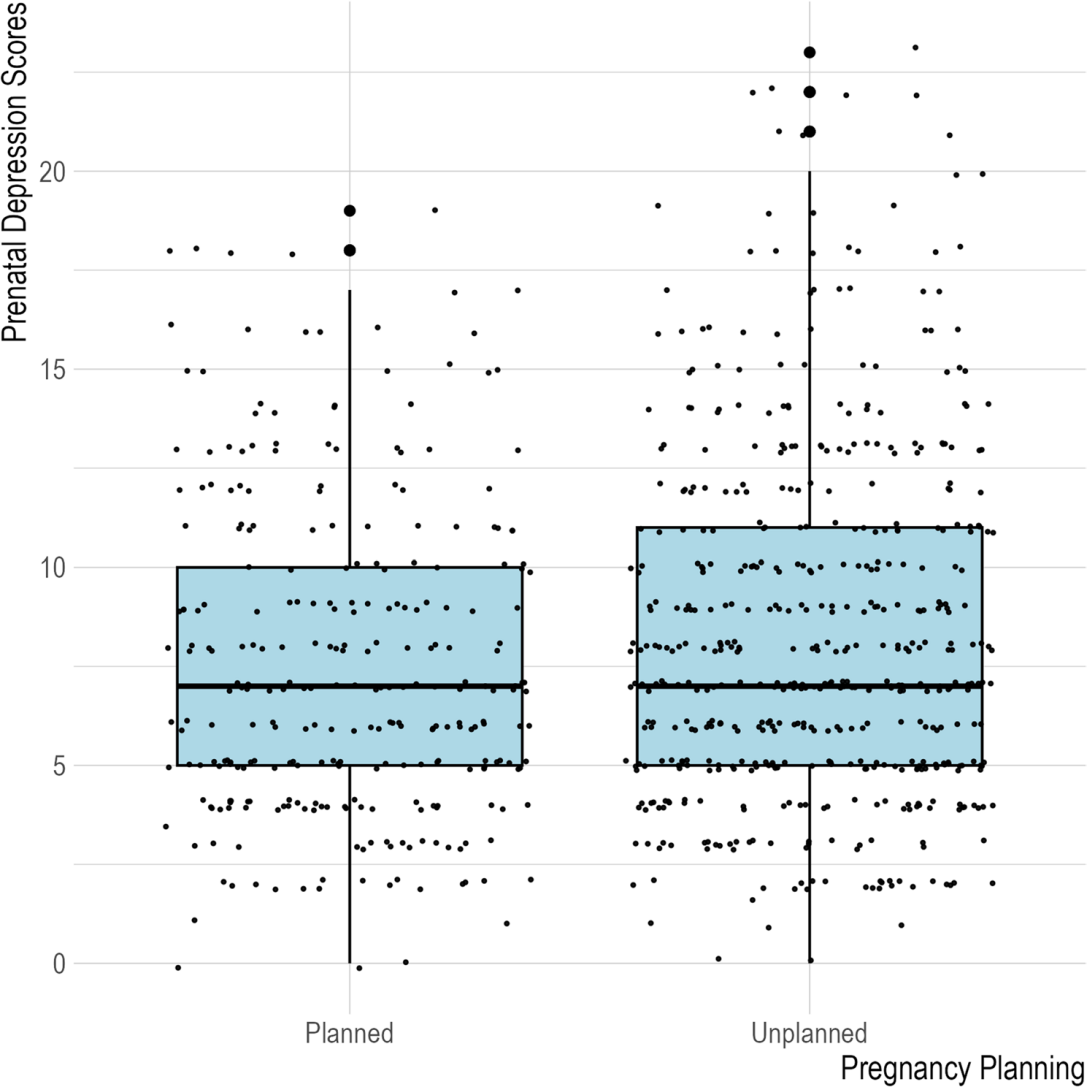
Distribution of Pregnancy Planning and Prenatal Depression Scores

Simple linear regression results suggested unplanned pregnancy was significantly associated with prenatal depression scores, such that those who identified their pregnancy as unplanned were more likely to report more severe depressive symptoms compared to those with planned pregnancies (β = 0.88; 95% CI [0.27, 1.49]; *p* = 0.005). Normality and homogeneity of variance were assessed by examining residual plots and other diagnostic tests of the simple linear model, and multicollinearity was assessed by examining the variance inflation factor in the multiple regressions. There was no evidence of assumption violations. Figure 1 provides a visualization of the data’s distribution.

Multiple linear regression results showed that the unplanned pregnancy variable was no longer significantly associated with prenatal depression scores after adjusting for the covariates of minoritized race/ethnicity, first-time parent status, language, education, and prenatal mental healthcare utilization (*p* = 0.435) (see Table 2). Model fit was shown with an adjusted R-squared = 0.13. None of the interaction terms evaluated were statistically significant (minoritized race/ethnicity interaction: *p* = 0.139; first-time parent status interaction: *p* = 0.683; education interaction: *p* = 0.397; prenatal mental healthcare utilization interaction: *p* = 0.912).

**Table 2:** Adjusted results for unplanned pregnancy in association with perinatal depression scores in full sample

Outcome	Unplanned Pregnancy Estimate	95% CI	*p*-value
Prenatal Depressive Symptoms	0.24	[−0.36, 0.84]	0.435
Postpartum Depressive Symptoms	−0.04	[−0.63, 0.56]	0.899

### Relationships among main study variables and postpartum depression in full sample

Multiple linear regression results showed that the unplanned pregnancy variable was not significantly associated with depression scores at 12 weeks postpartum when adjusting for the covariates of prenatal depression scores, minoritized race/ethnicity, first-time parent status, language, education, and prenatal mental healthcare utilization (*p* = 0.899) (see Table 2). Model fit was shown with an adjusted R-squared = 0.32. None of the interaction terms evaluated were statistically significant (minoritized race/ethnicity interaction: *p* = 0.319; first-time parent status interaction: *p* = 0.952; education interaction: *p* = 0.487; prenatal mental healthcare utilization interaction: *p* = 0.596).

### Subgroup analyses

#### Latine subgroup: relationships between unplanned pregnancy and prenatal depression

**Fig. 2:**
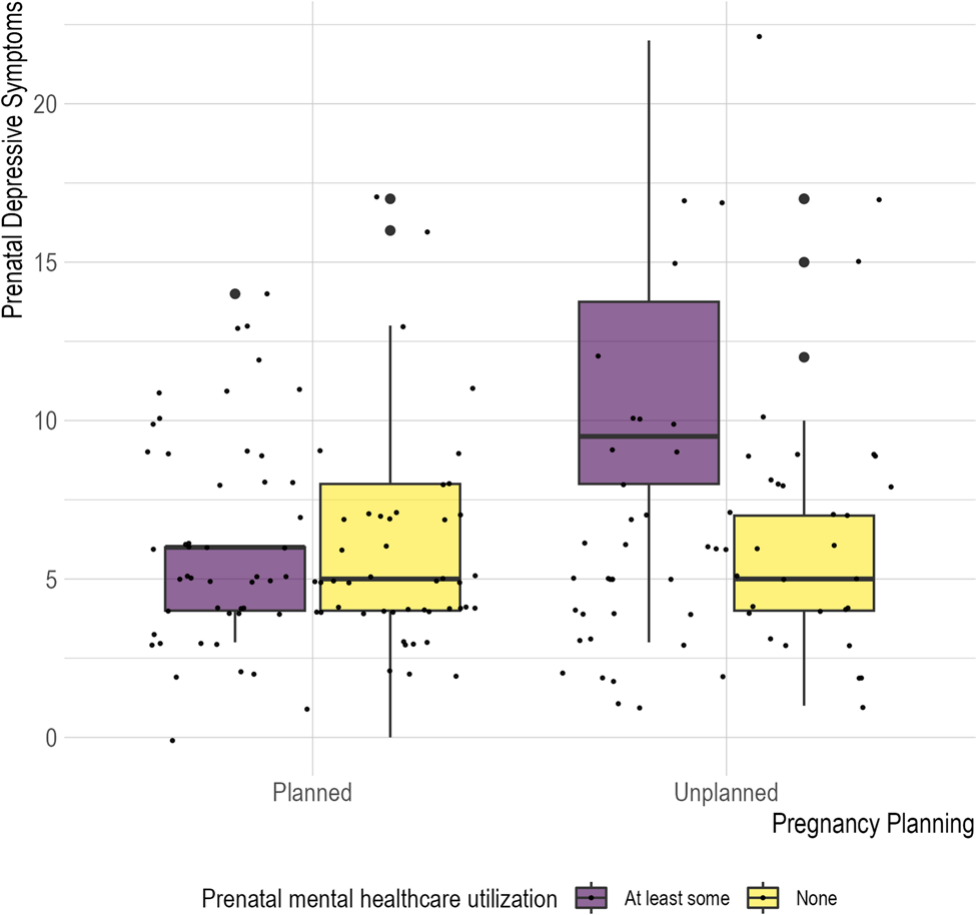
Unplanned pregnancy and prenatal mental healthcare utilization interaction associated with prenatal depression among Latine participants

When evaluating the association between unplanned pregnancy and prenatal depressive symptoms with our pre-specified covariates in the Latine subgroup, unplanned pregnancy was not significantly associated with prenatal depression scores (*p* = 0.868) (see Table 3). There was a significant interaction between pregnancy planning and prenatal mental healthcare utilization (β = 4.59, 95% CI [0.60, 8.59], *p* = 0.025), in that those in the Latine subgroup with unplanned pregnancy and prenatal mental healthcare utilization had more severe prenatal depressive symptoms compared to those in the Latine subgroup who did not endorse unplanned pregnancy or prenatal mental healthcare utilization (see Fig. 2). Beyond this, none of the other interaction terms evaluated were statistically significant (first-time parent status interaction: *p* = 0.546; education interaction: *p* = 0.233).

#### Latine subgroup: relationships between unplanned pregnancy and postpartum depression

When considering depressive symptoms at 12 weeks postpartum in the Latine subgroup, unplanned pregnancy was associated with more severe depression scores (β = 1.06; 95% CI [0.10, 2.03]; *p* = 0.031) compared to those who endorsed their pregnancies as planned when adjusting for the pre-specified covariates (see Table 3). None of the interaction terms evaluated were statistically significant (first-time parent status interaction: *p* = 0.494; education interaction: *p* = 0.405; prenatal mental healthcare utilization interaction: *p* = 0.681).

**Table 3:** Adjusted Results for Unplanned Pregnancy in Association with Perinatal Depression Scores in Racial/Ethnic Subgroups

**Racial/Ethnic Subgroup**	**Outcome**	**Estimate**	**95% CI**	***p*** **-value**
Latine	Prenatal Depressive Symptoms	0.10	[-1.07, 1.26]	0.868
	Postpartum Depressive Symptoms	1.06	[0.10, 2.03]	0.031
Black/African American	Prenatal Depressive Symptoms	-0.58	[-1.60, 0.44]	0.263
	Postpartum Depressive Symptoms	-0.21	[-1.27, 0.86]	0.699
White	Prenatal Depressive Symptoms	0.88	[-0.17, 1.93]	0.099
	Postpartum Depressive Symptoms	-0.71	[-1.72, 0.30]	0.165

#### Black/African American subgroup: relationships between unplanned pregnancy and prenatal depression

In the Black/African American subgroup, pregnancy planning was not significantly associated with prenatal depression symptoms when controlling for the pre-specified covariates (*p* = 0.263) (see Table 3). Additionally, none of the interaction terms were significant (first-time parent status interaction: *p* = 0.391; education interaction: *p* = 0.365); however, the interaction between pregnancy planning and prenatal mental healthcare utilization was trending towards significance, in that those who endorsed planned pregnancies and prenatal mental healthcare utilization had more severe depressive symptoms compared to those with unplanned pregnancies and prenatal mental health utilization (β = −2.48, 95% CI [−5.01, 0.05], *p* = 0.055).

#### Black/African American subgroup: relationships between unplanned pregnancy and postpartum depression

In the Black/African American subgroup, the unplanned pregnancy variable was not significantly associated with depression scores at 12 weeks postpartum when controlling for the pre-specified covariates (*p* = 0.699) (see Table 3). None of the interaction terms evaluated were statistically significant (first-time parent status interaction: *p* = 0.276; education interaction: *p* = 0.976; prenatal mental healthcare utilization interaction: *p* = 0.436).

#### White subgroup: relationships between unplanned pregnancy and prenatal depression

Unlike in the Latine and Black/African American subgroups, in the unadjusted model, unplanned pregnancy was significantly related to more severe prenatal depression scores in the White subgroup when comparing those whose pregnancies were planned (β = 1.34; 95% CI [0.27, 2.41], *p* = 0.014). However, after controlling for education, first-time parent status, prenatal mental healthcare utilization, and language, unplanned pregnancy was no longer significantly associated with prenatal depression scores in the White subgroup (*p* = 0.099) (see Table 3). None of the interaction terms evaluated were statistically significant (first-time parent status interaction: *p* = 0.490; education interaction: *p* = 0.620; prenatal mental healthcare utilization interaction: *p* = 0.135).

#### White subgroup: relationships between unplanned pregnancy and postpartum depression

Unplanned pregnancy was not significantly associated with depression scores at 12 weeks postpartum in the White subgroup in the adjusted model (*p* = 0.165) (see Table 3). None of the interaction terms evaluated were statistically significant (first-time parent status interaction: *p* = 0.172; education interaction: *p* = 0.189; prenatal mental healthcare utilization interaction: *p* = 0.867).

## Discussion

The present study sought to better understand how unplanned pregnancy may be associated with perinatal depression in a racially and ethnically diverse and low-income sample of birthing people in the United States. Due to systems of oppression, these populations are more likely to experience unplanned pregnancy and perinatal depression, as well as a variety of additional stressors, such as racial/ethnic discrimination at individual and systems levels, economic marginalization, and a lack of access to culturally responsive physical and mental healthcare, among others [15, 19, 38, 39]. Therefore, it is critical to understand how unplanned pregnancy may be associated with perinatal depression in the unique context of these populations.

Briefly, when looking at the full sample, unplanned pregnancy was associated with prenatal depressive symptoms in the unadjusted model; however, unplanned pregnancy was not found to have a main effect on prenatal or postpartum depressive symptoms when the model was adjusted for covariates. Different trends were found among racial/ethnic subgroups. Specifically, those in the Latine subgroup who had endorsed prenatal mental healthcare utilization and unplanned pregnancy had more severe prenatal depressive symptoms than those who endorsed their pregnancies as planned. Additionally in the Latine subgroup, unplanned pregnancy was associated with more severe depressive symptoms at 12 weeks postpartum compared to those with planned pregnancies.

The lack of association between unplanned pregnancy and perinatal depressive symptoms in the present study’s full sample differs from other research that has found an association between unplanned pregnancy and maternal depression [5, 7]. However, the present study adds to a small literature base that explores the relationship between unplanned pregnancy and perinatal depression in a racially and ethnically diverse and low-income sample. Indeed, among the few studies that have evaluated pregnancy planning and perinatal depression in racially diverse and low-income samples, the findings have been mixed [40–42]. For example, when adjusting for covariates and psychosocial characteristics, Maxson et al. did not find that prenatal depression was significantly associated with unwanted or mistimed pregnancy in a sample that was 77% non-Hispanic Black, with 51% of participants reporting annual income below $20,000 [41]. Additionally, Suh et al. found that among their sample, which was 40% Black and predominantly low-income, pregnancy intention did not predict mild nor severe postpartum depressive symptoms among the Black subgroup, but this association existed among the White subgroup [42]. However, Cruz-Bendezú did find an association between unintended pregnancy and prenatal depressive symptoms in their sample, which was primarily low-income and 50% Latine [40].

When considering the present study’s findings and the extant literature together, perhaps unplanned pregnancy may not be a particularly salient driver of perinatal depression in racially and ethnically diverse and low-income populations. It is possible that unplanned pregnancy is too distal of a stressor compared to other, more proximal concerns, such as experiences of financial insecurity and/or minority stress in being associated with perinatal depression. Moreover, it is possible that unique protective factors, such as collective social support, may buffer against the adverse mental health impacts of unplanned pregnancy in these populations [12, 43, 44]. This warrants further investigation.

When stratifying by racial/ethnic subgroups, unplanned pregnancy was found to be associated with perinatal depression in certain contexts in the Latine subgroup, highlighting the importance of evaluating unplanned pregnancy’s impact on perinatal depression within individual racial and ethnic subgroups, which has not previously been given sufficient attention in the literature. Systemic factors likely explain why Latine participants who endorsed unplanned pregnancy and prenatal mental healthcare utilization had greater prenatal depressive symptoms compared to those who endorsed planned pregnancies. There is evidence that communities minoritized by race and ethnicity utilize mental health services at lower rates compared to White populations due to a lack of culturally responsive care, the perpetuation of harm committed by healthcare providers against minoritized communities, the inaccessibility of comprehensive health insurance coverage, and high rates of stigma [38]. Further, there is evidence that Latine individuals are less likely to have health insurance compared to Black/African American individuals, potentially due to language and cultural factors, as well as high levels of poverty, especially among recent immigrants [45, 46]. Therefore, it is possible that Latine individuals in this sample who initiated psychotherapy and/or a medication prescription for depression may have experienced more baseline mental health challenges, which were exacerbated in the context of an unplanned pregnancy.

More research is needed to understand the mechanisms behind this finding though, especially given that in the Black/African American subgroup, there was a marginally significant interaction in which prenatal mental healthcare utilization and unplanned pregnancy were associated with *less* severe prenatal depressive symptoms compared to those with prenatal mental healthcare utilization and planned pregnancy. These discrepant findings, which were not found prior to stratifying the sample by race and ethnicity, reinforce the need to conduct research with racially and ethnically diverse samples and to evaluate unique contextual and protective factors in each group.

Further, while pregnancy planning did not have a main effect on prenatal depression symptoms in the Latine group, unplanned pregnancy was associated with more severe depression symptoms at 12 weeks postpartum compared to those who endorsed their pregnancies as planned in the Latine subgroup. A similar trend was observed in a study published by Christensen et al. (2011), in which the trajectory of depression symptoms across the prenatal and postpartum periods were analyzed in a sample of low-income, Hispanic immigrants. In this sample, unintended pregnancy was associated with the “Postpartum High” trajectory, meaning that depressive symptoms were borderline during the prenatal period, but then saw a substantial increase during the postpartum period [47]. These findings highlight the potential downstream effects of unplanned pregnancy later in the perinatal period and the need to provide support to postpartum birthing persons, even if they may not exhibit elevated prenatal depressive symptoms. Based on this study and the study from Christensen et al. (2011), these findings may be particularly salient in Latine populations.

Overall, unplanned pregnancy appeared to be associated with more severe perinatal depressive symptoms under certain conditions in the Latine subgroup, but not in other racial/ethnic subgroups in this sample. Therefore, the findings from the present study suggest that Latine individuals whose pregnancies are unplanned may benefit from additional screening and mental health support for depression throughout the perinatal period. These analyses provide a foundation for exploring mechanistic explanations for these findings, as well as protective factors, among Latine birthing people. Given that unplanned pregnancy was not consistently associated with perinatal depressive symptoms throughout all subgroups, perhaps interventions that increase institutionalized support for birthing people who experience minority stress and financial insecurity (e.g., paid parental leave, more social safety net measures, accessible and culturally-responsive mental healthcare) may be most beneficial in buffering against perinatal depression in these populations broadly.

It is important to note that these data were gathered prior to the 2022 Supreme Court ruling, Dobbs v. Jackson Health Center, which ruled that the constitutional right to an abortion is no longer federally protected in the United States, providing individual states with the opportunity to outlaw abortion. As of July 16, 2025, twelve states in the United States have implemented a total ban on abortion, and an additional four states have banned abortion after six weeks [48]. There are robust findings that those who are low-income and who hold racially and ethnically minoritized identities are among the most likely to be adversely impacted by abortion bans [49]. Further, research has shown that those who are denied abortion care due to restrictions experience heightened symptoms of anxiety [50]. Therefore, in the wake of a restrictive and volatile abortion landscape in the United States, access to abortion care should be considered a critical contextual factor when studying unplanned pregnancy and its associations with mental health, especially among low-income and racially and ethnically diverse populations.

## Limitations

The current study is not without limitations. First, intention of pregnancy was assessed with the single, dichotomous question of, “Was your current pregnancy planned,” which may not adequately reflect the complexity of pregnancy intention [51]. Additional constructs beyond lack of planning, such as poor timing and ambivalent or negative emotions associated with the pregnancy, have been shown to be associated with worse mental health outcomes than those that simply measure planning on its own [12, 52]. However, Barton et al. found increased odds of psychological distress among those who reported an unplanned pregnancy, even when they reported positive feelings associated with the pregnancy [12].

Additionally, due to subgroup size constraints, we were only able to conduct subgroup analyses on Black/African American, Latine, and White subgroups. This excludes several racial/ethnic groups, including Asian American, Pacific Islander, and Indigenous birthing persons. These groups may be at increased risk for perinatal depression [53]. These analyses were further limited by data collection only permitting participants to select a single racial identity or “Bi-racial,” which erases the nuance and complexity of multiracial identities from these analyses.

Finally, since this was a secondary analysis of data from a study with a different overall goal, it inherently carries biases. There were no formal power/sample size calculations completed for these analyses a priori.

## Future directions

Future research should prioritize the use of a more comprehensive pregnancy planning measure, one that assesses timing, desire, and quality of relationship with partner if applicable [51], in racially and ethnically diverse and low-income samples. Additionally, future studies should utilize a robust measure of pre-pregnancy mental health that explicitly asks about behaviors prior to pregnancy in order to better control for the effect of pre-pregnancy depression.

Moreover, studies should evaluate the trajectories of the association between pregnancy planning and depressive symptoms throughout the perinatal period with measurements conducted at multiple timepoints during the prenatal and postpartum periods. Individuals in low-income and racially and ethnically diverse populations may face a variety of stressors and protective factors over the course of the perinatal period that may influence how pregnancy planning impacts their experience of depression.

It is possible there are additional potential moderators that were not included in our models (e.g., experiences of discrimination, justified medical distrust, perceived support, perceived stress, access to reproductive healthcare, income/SES, trauma history, age, etc.), that could play a role in the relationship between unplanned pregnancy and perinatal depression. Even though unplanned pregnancy was not associated with depressive scores on its own with covariates included in our models, there may be other variables that it does interact with that were not assessed. Further, future studies must look beyond identity-based factors to understand what systemic factors account for differences based on identities. It cannot be emphasized strongly enough that differences based on demographic characteristics are the consequence of systems of oppression, not inherent to the identities themselves.

## Conclusions

The current study addressed important gaps in the literature by evaluating the association between pregnancy planning and perinatal depression in a racially and ethnically diverse and low-income sample. Analyses revealed that unplanned pregnancy may not be a particularly salient predictor of perinatal depression in this sample as a whole; however, subgroup analyses suggested that unplanned pregnancy was associated with higher perinatal depressive symptoms under certain conditions in the Latine subgroup. Future research should acknowledge the myriad stressors and protective factors experienced by racially and ethnically diverse and low-income perinatal populations, as well as continue to study differences in outcomes among racial/ethnic subgroups to capture important nuance.

## Supplementary Information

Supplementary Material 1. Relationships among Main Study Variables and Pregnancy Planning. Description of data: We conducted a post hoc analysis involving a binary logistic regression to examine the impact of baseline covariates on predicting unplanned pregnancy in the full sample.

Supplementary Material 2. Relationships among Main Study Variables and Pregnancy Planning in Full Sample and Racial and Ethnic Subgroups. Full results of the multiple linear regression models from the full sample and subgroup analyses by racial and ethnic subgroups.

## Data Availability

Upon reasonable request, the final dataset will be de-identified for sharing with members of the research community to advance science and health. We will remove or code any personal information that could identify participants before files are shared with other researchers to ensure that, by current scientific standards and known methods, no one will be able to identify these participants from the information we share.

## References

[1] Woody CA, Ferrari AJ, Siskind DJ, Whiteford HA, Harris MG. A systematic review and meta-regression of the prevalence and incidence of perinatal depression. J Affect Disord. 2017;219:86–92. 28531848 10.1016/j.jad.2017.05.003

[2] McKee K, Admon LK, Winkelman TNA, Muzik M, Hall S, Dalton VK, et al. Perinatal mood and anxiety disorders, serious mental illness, and delivery-related health outcomes, united states, 2006–2015. BMC Womens Health. 2020;20(1):150. 32703202 10.1186/s12905-020-00996-6 PMC7376899

[3] Goodman JH. Perinatal depression and infant mental health. Arch Psychiatr Nurs. 2019;33(3):217–24. 31227073 10.1016/j.apnu.2019.01.010

[4] Tirumalaraju V, Suchting R, Evans J, Goetzl L, Refuerzo J, Neumann A, et al. Risk of depression in the adolescent and adult offspring of mothers with perinatal depression: A systematic review and Meta-analysis. JAMA Netw Open. 2020;3(6):e208783. 32602910 10.1001/jamanetworkopen.2020.8783 PMC7327545

[5] Abajobir AA, Maravilla JC, Alati R, Najman JM. A systematic review and meta-analysis of the association between unintended pregnancy and perinatal depression. J Affect Disord. 2016;192:56–63. 26707348 10.1016/j.jad.2015.12.008

[6] Rossen L, Hamilton EB, Abma J, Gregory EC, Beresovsky V, Resendez A, Chandra A, Martin JA. Updated Methodology to Estimate Overall and Unintended Pregnancy Rates in the United States [Internet]. National Center for Health Statistics (U.S.); 2023. [cited 2024 Nov 14]. Available from: https://stacks.cdc.gov/view/cdc/124395

[7] Nelson HD, Darney BG, Ahrens K, Burgess A, Jungbauer RM, Cantor A, et al. Associations of unintended pregnancy with maternal and infant health outcomes: A systematic review and Meta-analysis. JAMA. 2022;328(17):1714. 36318133 10.1001/jama.2022.19097 PMC9627416

[8] Steinberg JR, Rubin LR. Psychological aspects of contraception, unintended pregnancy, and abortion. Policy Insights Behav Brain Sci. 2014;1(1):239–47. 25938133 10.1177/2372732214549328 PMC4416399

[9] Bedaso A, Adams J, Peng W, Sibbritt D. The relationship between social support and mental health problems during pregnancy: a systematic review and meta-analysis. Reprod Health. 2021;18(1):162. 34321040 10.1186/s12978-021-01209-5 PMC8320195

[10] Collins NL, Dunkel-Schetter C, Lobel M, Scrimshaw SCM. Social support in pregnancy: Psychosocial Correlates of Birth Outcomes and Postpartum Depression. J Pers Soc Psychol. 1993;65(6):1243. 10.1037//0022-3514.65.6.1243 8295121

[11] Lundsberg LS, Cutler AS, Stanwood NL, Yonkers KA, Gariepy AM. Association of pregnancy contexts with depression and low social support in early pregnancy. Perspect Sex Reprod Health. 2020;52(3):161–70. 33047499 10.1363/psrh.12155

[12] Barton K, Redshaw M, Quigley MA, Carson C. Unplanned pregnancy and subsequent psychological distress in partnered women: a cross-sectional study of the role of relationship quality and wider social support. BMC Pregnancy Childbirth. 2017;17(1):44. 28122585 10.1186/s12884-017-1223-x PMC5267424

[13] Hall KS, Kusunoki Y, Gatny H, Barber J. The risk of unintended pregnancy among young women with mental health symptoms. Soc Sci Med. 2014;100:62–71. 24444840 10.1016/j.socscimed.2013.10.037 PMC3898511

[14] Stidham Hall K, Moreau C, Trussell J, Barber J. Young women’s consistency of contraceptive use — does depression or stress matter? Contraception. 2013;88(5):641–9. 23850075 10.1016/j.contraception.2013.06.003 PMC3796023

[15] Kim TY, Dagher RK, Chen J. Racial/ethnic differences in unintended pregnancy. Am J Prev Med. 2016;50(4):427–35. 26616306 10.1016/j.amepre.2015.09.027

[16] Mukherjee S, Trepka MJ, Pierre-Victor D, Bahelah R, Avent T. Racial/Ethnic disparities in antenatal depression in the united states: A systematic review. Matern Child Health J. 2016;20(9):1780–97. 27016352 10.1007/s10995-016-1989-x

[17] Liu CH, Tronick E. Rates and predictors of postpartum depression by race and ethnicity: results from the 2004 to 2007 new York City PRAMS survey (Pregnancy risk assessment monitoring System). Matern Child Health J. 2013;17(9):1599–610. 23095945 10.1007/s10995-012-1171-z

[18] Horowitz JM, Igielnik R, Kochhar R. Most Americans say there is too much economic inequality in the U.S., but fewer than half call it a top priority. Pew Research Center; 2020;9:1340-65.

[19] Wishart D, Cruz Alvarez C, Ward C, Danner S, O’Brian CA, Simon M. Racial and ethnic minority pregnant patients with low-income experiences of perinatal care: a scoping review. Health Equity. 2021;5(1):554–68. 34909522 10.1089/heq.2021.0017 PMC8665802

[20] Piston S, Johnson KR, Hedlund S, Walker C. The Study of Racism and Policing in the United States.Annu Rev Polit Sci. 2025;28.

[21] Haight SC, Daw JR, Martin CL, Sheffield-Abdullah K, Verbiest S, Pence BW, et al. Racial and ethnic inequities in postpartum depressive symptoms, diagnosis, and care in 7 US jurisdictions: study examines racial and ethnic inequities in postpartum depressive symptoms, diagnosis, and care. Health Aff (Millwood). 2024;43(4):486–95. 38560804 10.1377/hlthaff.2023.01434 PMC13034582

[22] Kozhimannil KB, Trinacty CM, Busch AB, Huskamp HA, Adams AS. Racial and ethnic disparities in postpartum depression care among low-income women. Psychiatr Serv .2011;62(6):619-25. 10.1176/ps.62.6.pss6206_0619. 10.1176/appi.ps.62.6.619 PMC3733216 21632730

[23] Fellenzer JL, Cibula DA. Intendedness of pregnancy and other predictive factors for symptoms of prenatal depression in a Population-Based study. Matern Child Health J. 2014;18(10):2426–36. 24752314 10.1007/s10995-014-1481-4

[24] Bradshaw H, Riddle JN, Salimgaraev R, Zhaunova L, Payne JL. Risk factors associated with postpartum depressive symptoms: a multinational study. J Affect Disord. 2022;301:345–51. 34979186 10.1016/j.jad.2021.12.121

[25] Míguez MC, Vázquez MB. Risk factors for antenatal depression: a review. World J Psychiatry. 2021;11(7):325–36. 34327125 10.5498/wjp.v11.i7.325 PMC8311510

[26] Underwood L, Waldie KE, D’Souza S, Peterson ER, Morton SMB. A longitudinal study of Pre-pregnancy and pregnancy risk factors associated with antenatal and postnatal symptoms of depression: evidence from growing up in new Zealand. Matern Child Health J. 2017;21(4):915–31. 27837388 10.1007/s10995-016-2191-x

[27] Jensen JK, Ciolino JD, Diebold A, Segovia M, Degillio A, Solano-Martinez J, et al. Comparing the effectiveness of clinicians and paraprofessionals to reduce disparities in perinatal depression via the mothers and babies course: protocol for a Cluster-Randomized controlled trial. JMIR Res Protoc. 2018;7(11):e11624. 30459138 10.2196/11624 PMC6280028

[28] Tandon SD, Johnson JK, Diebold A, Segovia M, Gollan JK, Degillio A, et al. Comparing the effectiveness of home visiting paraprofessionals and mental health professionals delivering a postpartum depression preventive intervention: a cluster-randomized non-inferiority clinical trial. Arch Womens Ment Health. 2021;24(4):629–40. 33655429 10.1007/s00737-021-01112-9

[29] Harris PA, Taylor R, Thielke R, Payne J, Gonzalez N, Conde JG. Research electronic data capture (REDCap)—A metadata-driven methodology and workflow process for providing translational research informatics support. J Biomed Inform. 2009;42(2):377–81. 18929686 10.1016/j.jbi.2008.08.010 PMC2700030

[30] Harris PA, Taylor R, Minor BL, Elliott V, Fernandez M, O’Neal L, et al. The REDCap consortium: Building an international community of software platform partners. J Biomed Inf. 2019;95:103208. 10.1016/j.jbi.2019.103208 PMC7254481 31078660

[31] Rush AJ, Trivedi MH, Ibrahim HM, Carmody TJ, Arnow B, Klein DN, et al. The 16-item quick inventory of depressive symptomatology (QIDS), clinician rating (QIDS-C), and self-report (QIDS-SR): a psychometric evaluation in patients with chronic major depression. Biol Psychiatry. 2003;54(5):573–83. 12946886 10.1016/s0006-3223(02)01866-8

[32] Reilly TJ, MacGillivray SA, Reid IC, Cameron IM. Psychometric properties of the 16-item quick inventory of depressive symptomatology: A systematic review and meta-analysis. J Psychiatr Res. 2015;60:132–40. 25300442 10.1016/j.jpsychires.2014.09.008

[33] Bernstein IH, Rush AJ, Yonkers K, Carmody TJ, Woo A, McConnell K, et al. Symptom features of postpartum depression: are they distinct? Depress Anxiety. 2008;25(1):20–6. 17187349 10.1002/da.20276 PMC2268615

[34] Call CC, Eckstrand KL, Kasparek SW, Boness CL, Blatt L, Jamal-Orozco N, et al. An ethics and social-justice approach to collecting and using demographic data for psychological researchers. Perspect Psychol Sci. 2023. 10.1177/17456916221137350. 36459692 10.1177/17456916221137350 PMC10235209

[35] Kornfield SL, Kang-Yi CD, Mandell DS, Epperson CN. Predictors and patterns of psychiatric treatment dropout during pregnancy among Low-Income women. Matern Child Health J. 2018;22(2):226–36. 29143169 10.1007/s10995-017-2394-9 PMC5821232

[36] Snapper LA, Hart KL, Venkatesh KK, Kaimal AJ, Perlis RH. Cohort study of the relationship between individual psychotherapy and pregnancy outcomes. J Affect Disord. 2018;239:253–7. 30029152 10.1016/j.jad.2018.05.083 PMC9980714

[37] Posit Team (2024). RStudio: Integrated Development Environment for R. Boston: Posit Software, PBC, Boston, MA. Available from: http://www.posit.co/.

[38] Maura J, Weisman De Mamani A. Mental health disparities, treatment engagement, and attrition among racial/ethnic minorities with severe mental illness: A review. J Clin Psychol Med Settings. 2017;24(3–4):187–210. 28900779 10.1007/s10880-017-9510-2

[39] Beech BM, Ford C, Thorpe RJ, Bruce MA, Norris KC. Poverty, racism, and the public health crisis in America. Front Public Health. 2021;9:699049. 34552904 10.3389/fpubh.2021.699049 PMC8450438

[40] Cruz-Bendezú AM, Lovell GV, Roche B, Perkins M, Blake-Lamb TL, Taveras EM, et al. Psychosocial status and prenatal care of unintended pregnancies among low-income women. BMC Pregnancy Childbirth. 2020;20(1):615. 33046003 10.1186/s12884-020-03302-2 PMC7552564

[41] Maxson P, Miranda ML. Pregnancy intention, demographic differences, and psychosocial health. J Womens Health. 2011;20(8):1215–23. 10.1089/jwh.2010.2379 21671765

[42] Suh EY, Ma P, Dunaway LF, Theall KP. Pregnancy intention and Post-partum depressive affect in Louisiana pregnancy risk assessment monitoring system. Matern Child Health J. 2016;20(5):1001–13. 26649877 10.1007/s10995-015-1885-9

[43] Sagrestano LM, Feldman P, Rini CK, Woo G, Dunkel-Schetter C. Ethnicity and social support during pregnancy. Am J Community Psychol. 1999;27(6):869–98. 10723538 10.1023/a:1022266726892

[44] Campos B, Schetter CD, Abdou CM, Hobel CJ, Glynn LM, Sandman CA. Familialism, social support, and stress: positive implications for pregnant Latinas. Cultur Divers Ethnic Minor Psychol. 2008;14(2):155–62. 18426288 10.1037/1099-9809.14.2.155 PMC2859297

[45] Hill L, Ndugga N, Artiga S, Damico A. Health coverage by race and ethnicity, 2010–2023. Kaiser Family Foundation; 2025. https://www.kff.org/racial-equity-and-health-policy/issue-brief/health-coverage-by-race-and-ethnicity/.

[46] Funk C, Lopez MH. Pew Research Center. Hispanic Americans’ trust in and engagement with science. Pew Research Center; 2022.

[47] Christensen AL, Stuart EA, Perry DF, Le HN. Unintended pregnancy and perinatal depression trajectories in low-income, high-risk Hispanic immigrants. Prev Sci. 2011;12(3):289–99. 21537899 10.1007/s11121-011-0213-x PMC3742050

[48] Guttmacher Institute. Interactive Map: US Abortion Policies and Access After Roe [Internet]. Guttmacher Institute. 2024. [cited 2024 Dec 5]. Available from: https://states.guttmacher.org/policies/idaho/abortion-policies.

[49] Axelson SM, Steiner RJ, Jones RK. Characteristics of U.S. Abortion patients who obtained care out of state prior to the overturning of roe v. Wade. Womens Health Issues. 2024;34(2):142–7. 38102056 10.1016/j.whi.2023.10.003

[50] Horvath S, Schreiber CA. Unintended, pregnancy. Induced abortion, and mental health. Curr Psychiatry Rep. 2017;19(11): 77. 28905259 10.1007/s11920-017-0832-4

[51] Santelli JS, Lindberg LD, Orr MG, Finer LB, Speizer I. Toward a multidimensional measure of pregnancy intentions: evidence from the united States. Stud Fam Plann. 2009;40(2):87–100. 19662801 10.1111/j.1728-4465.2009.00192.x

[52] Gariepy AM, Lundsberg LS, Miller D, Stanwood NL, Yonkers KA. Are pregnancy planning and pregnancy timing associated with maternal psychiatric illness, psychological distress and support during pregnancy? J Affect Disord. 2016;205:87–94. 27423065 10.1016/j.jad.2016.06.058 PMC5048515

[53] Miller ML, Dupree J, Monette MA, Lau EK, Peipert A. Health equity and perinatal mental health. Curr Psychiatry Rep. 2024;26(9):460–9. 39008146 10.1007/s11920-024-01521-4

