# Supplementary material for: Unplanned pregnancy and perinatal depression: secondary exploratory analyses from a racially and ethnically diverse, low-income sample of birthing people in the United States
Source: BMC Pregnancy Childbirth. 2025 Aug 21;25:870. doi: 10.1186/s12884-025-08009-w (PMC12369065; doi:10.1186/s12884-025-08009-w)
Supplement: Supplementary file 1 — Supplementary Material 1. Relationships among Main Study Variables and Pregnancy Planning. Description of data: We conducted a post hoc analysis involving a binary logistic regression to examine the impact of baseline covariates on predicting unplanned pregnancy in the full sample. [file 12884_2025_8009_MOESM1_ESM.docx]

**Additional File 1:** Relationships among Main Study Variables and Pregnancy Planning

We conducted a post hoc analysis involving a binary logistic regression to examine the impact of baseline covariates on unplanned pregnancy in the full sample.

**Table 12:** Logistic Regression Analysis Results for Unplanned Pregnancy

| **Variable** | **Estimate (Odds Ratio)** | **Lower 95% Confidence Limit** | **Upper 95% Confidence Limit** | **p-value** |
| --- | --- | --- | --- | --- |
| Minoritized race/ethnicity | 1.73 | 1.23 | 2.44 | 0.002 |
| First-time parent status | 1.78 | 1.29 | 2.48 | <0.001 |
| Spanish Language | 0.23 | 0.14 | 0.36 | <0.001 |
| Some College Education | 0.56 | 0.41 | 0.76 | <0.001 |
| Prenatal mental healthcare utilization | 1.19 | 0.79 | 1.81 | 0.415 |

As we have noted, over the course of the analyses, the variation in depressive symptoms explained by the pre-specified covariates seemed to outweigh that explained by pregnancy planning alone in the full sample. Therefore, the question arose regarding true confounding. As a result, we conducted an exploratory post hoc logistic regression analysis with planned pregnancy as the outcome and those covariates as potential predictors. **Table 12** illustrates these analytic results, and we see that minoritized race/ethnicity, first-time parent status, language, and education are all significantly associated with pregnancy planning in this sample. Those whose primary language was Spanish and those with at least some college education were more likely to report a planned pregnancy, and first-time parents and those with minoritized race/ethnicity were more likely to report an unplanned pregnancy. The Hosmer-Lemeshow Goodness of Fit test did not suggest evidence of a poor model fit.

These post hoc analyses revealed similarities between the risk factors for unplanned pregnancy and the risk factors for perinatal depression in this sample. This suggests that those who are at risk for unplanned pregnancy may also be at risk for perinatal depression.
