# Supplementary material for: Unplanned pregnancy and perinatal depression: secondary exploratory analyses from a racially and ethnically diverse, low-income sample of birthing people in the United States
Source: BMC Pregnancy Childbirth. 2025 Aug 21;25:870. doi: 10.1186/s12884-025-08009-w (PMC12369065; doi:10.1186/s12884-025-08009-w)
Supplement: Supplementary file 2 — Supplementary Material 2. Relationships among Main Study Variables and Pregnancy Planning in Full Sample and Racial and Ethnic Subgroups. Full results of the multiple linear regression models from the full sample and subgroup analyses by racial and ethnic subgroups. [file 12884_2025_8009_MOESM2_ESM.docx]

**Additional file 2**: Relationships among Main Study Variables and Pregnancy Planning in Full Sample and Racial and Ethnic Subgroups

***Full Sample***

**Table 4:** Multiple Linear Regression Model Results for Prenatal Depression Scores in Full Sample

| **Variable** | **Estimate** | **Lower 95% Confidence Limit** | **Upper 95% Confidence Limit** | **p-value** |
| --- | --- | --- | --- | --- |
| Unplanned pregnancy | 0.24 | -0.36 | 0.84 | 0.435 |
| Minoritized race/ethnicity | 1.30 | 0.67 | 1.92 | <0.001 |
| First-time parent status | 0.79 | 0.21 | 1.37 | 0.007 |
| Spanish Language | -2.17 | -3.03 | -1.31 | <0.001 |
| Some College Education | 0.19 | -0.38 | 0.76 | 0.510 |
| Prenatal mental healthcare utilization | 3.21 | 2.48 | 3.95 | <0.001 |

**Table 4** illustrates the results of multiple linear regression. While a number of the prespecified covariates were statistically significant (minoritized race/ethnicity, first-time parent status, English language, prenatal mental healthcare utilization), the unplanned pregnancy variable was no longer significantly associated with prenatal depression scores after adjusting for these important predictors. Model fit was shown with an adjusted R-squared = 0.13. None of the interaction terms evaluated were statistically significant (minoritized race/ethnicity interaction: *p* = 0.139; first-time parent status interaction: *p* = 0.683; education interaction: *p* = 0.397; prenatal mental healthcare utilization interaction: *p* = 0.912).

**Table 5:** Multiple Linear Regression Results for 12-Week Postpartum Depression Score in Full Sample

| **Variable** | **Estimate** | **Lower 95% Confidence Limit** | **Upper 95% Confidence Limit** | **p-value** |
| --- | --- | --- | --- | --- |
| Unplanned pregnancy | -0.04 | -0.63 | 0.56 | 0.899 |
| Prenatal depression scores | 0.55 | 0.48 | 0.62 | <0.001 |
| Minoritized race/ethnicity | -0.66 | -1.29 | -0.03 | 0.041 |
| Some College Education | 0.61 | 0.05 | 1.18 | 0.033 |
| First-time parent status | -0.66 | -1.24 | -0.07 | 0.028 |
| Prenatal mental healthcare utilization | 1.43 | 0.64 | 2.21 | <0.001 |
| Spanish Language | -0.41 | -1.26 | 0.44 | 0.347 |

**Table 5** illustrates the results of multiple linear regression for the 12-week postpartum depressive symptom scores. While several of the prespecified covariates were statistically significant (prenatal depression scores, minoritized race/ethnicity, at least some college education, first-time parent status, and prenatal mental healthcare utilization), the unplanned pregnancy variable was not significantly associated with postpartum depression scores in this model. Model fit was shown with an adjusted R-squared = 0.32. None of the interaction terms evaluated were statistically significant (minoritized race/ethnicity interaction: *p* = 0.319; first-time parent status interaction: *p* = 0.952; education interaction: *p* = 0.487; prenatal mental healthcare utilization interaction: *p* = 0.596).

***Racial/Ethnic Subgroup Analyses:***

**Table 6:** Multiple Linear Regression Results for Prenatal Depression Scores in Latine subgroup

| **Variable** | **Estimate** | **Lower 95% Confidence Limit** | **Upper 95% Confidence Limit** | **p-value** |
| --- | --- | --- | --- | --- |
| Unplanned pregnancy | 0.10 | -1.07 | 1.26 | 0.868 |
| Some College Education | 1.38 | 0.21 | 2.55 | 0.021 |
| First-time parent status | 0.19 | -1.15 | 1.53 | 0.780 |
| Prenatal mental healthcare utilization | 2.99 | 0.98 | 4.99 | 0.004 |
| Spanish Language | -0.89 | -2.12 | 0.34 | 0.153 |

**Table 6** illustrates the results of multiple linear regression for the prenatal depressive symptom scores in the Latine subgroup. While some of the prespecified covariates were statistically significant (education and prenatal mental healthcare utilization), the unplanned pregnancy variable was not significantly associated with prenatal depression scores in this model. Model fit was shown with an adjusted R-squared value of 0.09.

**Table 7:** Multiple Linear Regression Results for 12 Weeks Postpartum Depression Scores in Latine subgroup

| **Variable** | **Estimate** | **Lower 95% Confidence Limit** | **Upper 95% Confidence Limit** | **p-value** |
| --- | --- | --- | --- | --- |
| Unplanned pregnancy | 1.06 | 0.10 | 2.03 | 0.031 |
| Prenatal QIDS scores | 0.56 | 0.43 | 0.70 | <0.001 |
| Some College Education | 0.77 | -0.21 | 1.76 | 0.123 |
| First-time parent status | -0.89 | -2.05 | 0.27 | 0.130 |
| Prenatal mental healthcare utilization | 0.58 | -1.14 | 2.30 | 0.509 |
| Spanish Language | -1.27 | -2.29 | -0.26 | 0.014 |

**Table 7** illustrates the results of multiple linear regression for the 12 weeks postpartum depressive symptom scores in the Latine subgroup. In this model, the unplanned pregnancy variable was significantly associated with depression scores at 12 weeks postpartum, in that those in the Latine subgroup whose pregnancies were unplanned had higher depressive symptoms than those in the Latine subgroup whose pregnancies were considered planned. Model fit was shown with an adjusted R-squared of 0.42.

**Table 8:** Multiple Linear Regression Results for Prenatal Depression Scores in Black/African American subgroup

| **Variable** | **Estimate** | **Lower 95% Confidence Limit** | **Upper 95% Confidence Limit** | **p-value** |
| --- | --- | --- | --- | --- |
| Unplanned pregnancy | -0.58 | -1.60 | 0.44 | 0.263 |
| Some College Education | -0.12 | -1.00 | 0.76 | 0.788 |
| First-time parent status | 0.958 | 0.09 | 1.83 | 0.031 |
| Prenatal mental healthcare utilization | 2.55 | 1.40 | 3.70 | <0.001 |
| Spanish Language | 2.51 | -3.19 | 8.23 | 0.387 |

**Table 8** illustrates the results of multiple linear regression for the prenatal depressive symptom scores in the Black/African American subgroup. While some of the prespecified covariates were statistically significant (first-time parent status and prenatal mental healthcare utilization), the unplanned pregnancy variable was not significantly associated with prenatal depression scores in this model. Model fit was shown with an adjusted R-squared value of 0.05.

**Table 9:** Multiple Linear Regression Results for 12 Weeks Postpartum Depression Scores in Black/African American subgroup

| **Variable** | **Estimate** | **Lower 95% Confidence Limit** | **Upper 95% Confidence Limit** | **p-value** |
| --- | --- | --- | --- | --- |
| Unplanned pregnancy | -0.21 | -1.27 | 0.86 | 0.699 |
| Prenatal QIDS scores | 0.44 | 0.33 | 0.56 | <0.001 |
| Some College Education | 1.04 | 0.11 | 1.98 | 0.029 |
| First-time parent status | -0.80 | -1.73 | 0.14 | 0.095 |
| Prenatal mental healthcare utilization | 2.35 | 1.04 | 3.65 | <0.001 |
| Spanish Language | 6.05 | 0.54 | 11.57 | 0.032 |

**Table 9** illustrates the results of multiple linear regression for the 12 weeks postpartum depressive symptom scores in the Black/African American subgroup. While several of the prespecified covariates were statistically significant (prenatal depression scores, education, Spanish language, and prenatal mental healthcare utilization), the unplanned pregnancy variable was not significantly associated with depression scores at 12 weeks postpartum in this model. Model fit was shown with an adjusted R-squared of 0.24.

**Table 10:** Multiple Linear Regression Results for Prenatal Depression Scores in White subgroup

| **Variable** | **Estimate** | **Lower 95% Confidence Limit** | **Upper 95% Confidence Limit** | **p-value** |
| --- | --- | --- | --- | --- |
| Unplanned pregnancy | 0.88 | -0.17 | 1.93 | 0.099 |
| Some College Education | 0.18 | -0.83 | 1.19 | 0.729 |
| First-time parent status | 0.72 | -0.29 | 1.71 | 0.159 |
| Prenatal mental healthcare utilization | 3.56 | 2.43 | 4.69 | <0.001 |
| Spanish Language | 3.14 | -4.55 | 10.83 | 0.422 |

Unlike in the Latine and Black/African American subgroups, simple linear regression results suggested unplanned pregnancy was significantly associated with prenatal depression scores in the White subgroup, such that those who identified their pregnancy as unplanned were more likely to report more severe depressive symptoms compared to those who had planned pregnancies (β = 1.34; 95% CI [0.27, 2.41], *p* = 0.014). **Table 10** illustrates the results of multiple linear regression for the prenatal depressive symptom scores in the White subgroup. While the covariate of prenatal mental healthcare utilization was statistically significant, the unplanned pregnancy variable was not significantly associated with prenatal depression scores after adjusting for these important predictors. Model fit was shown with an adjusted R-squared value of 0.15.

**Table 11:** Multiple Linear Regression Results for 12 Weeks Postpartum Depression Scores in White subgroup

| **Variable** | **Estimate** | **Lower 95% Confidence Limit** | **Upper 95% Confidence Limit** | **p-value** |
| --- | --- | --- | --- | --- |
| Unplanned pregnancy | -0.71 | -1.72 | 0.30 | 0.165 |
| Prenatal depression scores | 0.75 | 0.62 | 0.88 | <0.001 |
| Some College Education | 0.21 | -0.77 | 1.19 | 0.673 |
| First-time parent status | -0.65 | -1.62 | 0.33 | 0.194 |
| Prenatal mental healthcare utilization | 0.22 | -0.98 | 1.42 | 0.717 |
| Spanish Language | -6.11 | -13.18 | 0.96 | 0.090 |

**Table 11** illustrates the results of multiple linear regression for the 12 weeks postpartum depressive symptom scores in the White subgroup. While prenatal depression scores were statistically significant, the unplanned pregnancy variable was not significantly associated with depression scores at 12 weeks postpartum. Model fit was shown with an adjusted R-squared of 0.42.
